# Enhancement in cellular Na^+^K^+^ATPase activity by low doses of peroxynitrite in mouse renal tissue and in cultured HK2 cells

**DOI:** 10.14814/phy2.12766

**Published:** 2016-04-14

**Authors:** Arpan K. Maiti, Mohammed T. Islam, Ryousuke Satou, Dewan S. A. Majid

**Affiliations:** ^1^ Department of PhysiologyHypertension and Renal Center of ExcellenceTulane University Health Sciences CenterNew OrleansLouisiana

**Keywords:** cellular Na^+^K^+^ATPase activity, HK2 cells, nitric oxide, Peroxynitrite, superoxide

## Abstract

In the normal condition, endogenous formation of peroxynitrite (ONOOˉ) from the interaction of nitric oxide and superoxide has been suggested to play a renoprotective role. However, the exact mechanism associated with renoprotection by this radical compound is not yet clearly defined. Although ONOOˉ usually inhibits renal tubular Na^+^K^+^ ATPase (NKA) activity at high concentrations (micromolar to millimolar range [*μ*M–mM], achieved in pathophysiological conditions), the effects at lower concentrations (nanomolar range [nM], relevant in normal condition) remain unknown. To examine the direct effect of ONOOˉ on NKA activity, preparations of cellular membrane fraction from mouse renal tissue and from cultured HK2 cells (human proximal tubular epithelial cell lines) were incubated for 10 and 30 min each with different concentrations of ONOOˉ (10 nmol/L–200 *μ*mol/L). NKA activity in these samples (*n* = 5 in each case) was measured via a colorimetric assay capable of detecting inorganic phosphate. At high concentrations (1–200 *μ*mol/L), ONOOˉ caused dose‐dependent inhibition of NKA activity (−3.0 ± 0.6% and −36.4 ± 1.4%). However, NKA activity remained unchanged at 100 and 500 nmol/L ONOOˉ concentration, but interestingly, at lower concentrations (10 and 50 nmol/L), ONOOˉ caused small but significant increases in the NKA activity (3.3 ± 1.1% and 3.1 ± 0.6%). Pretreatment with a ONOOˉ scavenger, mercaptoethylguanidine (MEG; 200 *μ*mol/L), prevented these biphasic responses to ONOOˉ. This dose‐dependent biphasic action of ONOO ^−^ on NKA activity may implicate that this radical compound helps to maintain sodium homeostasis either by enhancing tubular sodium reabsorption under normal conditions or by inhibiting it during oxidative stress conditions.

## Introduction

Peroxynitrite (ONOO^−^) is a reactive nitrogen species that is produced in biological systems when nitric oxide (NO) and superoxide (O_2_^.−^) react nonenzymatically at a near equimolar ratio (Pacher et al. 2007; Szabó et al. 2007). Despite the short half‐life at physiological pH, ONOO^−^ generated from a cellular source can influence surrounding target cells within one to two cell diameters (Marla et al. 1997; Denicola et al. 1998; Szabó et al. 2007). ONOO^−^ can induce biological effects influencing cellular reactions such as the initiation of lipid peroxidation (Katz 1982; King et al. 1992; Szabo et al. 1997), inhibition of mitochondrial respiration (Hausladen and Fridovich 1994; Radi et al. 1994; Boldyrev et al. 1997; Szabo et al. 1997), inhibition of membrane pumps (Hu et al. 1994), depletion of antioxidant enzymes like glutathione (Quijano et al. 2005), and damage to DNA (King et al. 1992; Inoue and Kawanishi 1995; Salgo et al. 1995; Szabo′ et al. 1996). These adverse effects occur mostly due to overproduction of ONOO^−^ in a variety of pathological conditions by direct or indirect oxidant mechanisms (Beckman and Koppenol 1996; Burney et al. 1999; Alvarez and Radi 2003; Matavelli et al. 2009). However, due to the presence of endogenous antioxidant defense mechanisms, the steady‐state concentrations of ONOO^−^ are estimated to be low in the nanomolar range (Radi et al. 2002; Nalwaya and Deen 2005; Quijano et al. 2005; Szabó et al. 2007; Matavelli et al. 2009), suggesting an importance of testing its effects at low concentrations in many biological functions.

Na^+^K^+^ATPase (NKA), located in the basolateral membranes of renal tubular cells, is the most important enzyme that establishes an electrochemical concentration gradient promoting the reabsorption of Na^+^ and other ions throughout the nephron (Katz 1982; Zhang et al. 2002).When the enzyme activity is almost completely abolished by maximal inhibitory concentrations of ouabain in the perfused rat kidney, about half of the fractional sodium reabsorption is inhibited; the rest has been linked to bicarbonate reabsorption and other processes that require metabolic energy (Katz 1982). Several lines of evidence from different studies in various tissue types, including the renal tissues, suggest that ONOO^−^ plays a role in regulating NKA activity and function (Zhang et al. 2002; Comellas et al. 2006; Reifenberger et al. 2008; White et al. 2008). (Zhang et al. (2002)) showed that in the proximal renal tubules, angiotensin II (Ang II) at picomolar concentrations stimulates NKA activity, but at nanomolar concentrations, inhibition of NKA occurs. In the same study (Zhang et al. 2002), it was also shown that at a particular concentration of 100 pmol/L (1 × 10^−7^ mol/L) of Ang II, the stimulatory effect on NKA was unmasked by scavengers of O_2_^.−^, NO, and ONOO^−^ indicating the direct involvement of O_2_^.−^, NO, and ONOO^−^ signaling in the regulation of NKA activity. However, Reifenberger et al. (Reifenberger et al. 2008) showed that when purified renal NKA is treated with a prolonged exposure of low concentrations of ONOO^−^ as might be produced intracellularly, NKA activity is inhibited. Thus, there remains much controversy about the effect of ONOO^−^ on NKA activity at lower concentrations in the physiological (nanomolar) range. An earlier in vivo study (Matavelli et al. 2009) conducted in anesthetized rats in our laboratory had demonstrated that an intra‐arterial administration of a low dose of ONOO^−^ (achieved physiologically more relevant plasma concentration) did not alter the urinary sodium excretion responses. These findings suggest that ONOO^−^ at a low concentration do not cause any inhibitory impact of on NKA in renal tubular cells, thus maintaining a constant level of sodium reabsorption throughout the nephron segments.

In the present study, we investigated the impact of various concentrations of ONOO^−^, starting at very low concentrations (low nanomolar [nM], physiologically relevant) and progressing to high concentrations (high micromolar [*μ*M], pathophysiologically relevant), in the regulation of NKA on renal plasma membrane fractions and on cultured human proximal tubular epithelial (HK2) cells. It is hypothesized that this a biphasic effect of ONOO^−^ on renal tubular NKA activity which is stimulated at low concentrations but inhibited at high concentrations of ONOO^−^. In these experiments, the specificity of these ONOO^−^‐induced responses was determined by using a specific ONOO^−^ scavenging agent, 2‐mercaptoethylguanidine sulfate (MEG) (Cuzzocrea et al. 1998). To determine whether superoxide (O_2_^−^) or nitric oxide (NO) has any direct role in the responses induced by ONOO^−^, the membrane fractions from the renal tissues and cultured HK2 cells were tested with or without cotreatment with TEMPOL (O_2_^−^ scavenger) and l‐NAME (NO synthase enzyme inhibitor) (Majid et al. 2005).

## Methods

### Preparation of ONOO^−^ solutions at different concentrations

Peroxynitrite (catalog no. 516620, Calbiochem) supplied at 1.1 mol/L NaOH was aliquoted and stored at −80°C. The exact concentration of ONOO^−^ was determined on the day of the experiment using the molar extinction coefficient of ONOO^−^ (1670/cm/mol/L). A thawed aliquot of 2.5 *μ*L of stock ONOO^−^ was diluted to 1 mL of 5 mol/L NaOH, and the absorbance was measured at 302 nmol/L. The calculated stock concentration of ONOO^−^ varied between 105 and 120 mmol/L. Determination of the concentration of stock ONOO^−^ allowed us to add the appropriate volume of stock ONOO^−^ (in 1.1 mol/L NaOH) to the samples at concentrations ranging from 10 nmol/L to 200 *μ*mol/L in isolated renal plasma membrane fractions and from 500 pmol/L to 200 *μ*mol/L in HK2 cells.

### Preparation of plasma membrane fraction from the mouse kidney

Mouse renal plasma membrane fractions were isolated following the method used by (Jorgensen et al. (1991)). Briefly, the kidneys collected from the sacrificed mice (mouse strain – C57BL6; 8–10 weeks old) were homogenized at 4°C in 5 mL of homogenizing buffer containing 0.25 mol/L sucrose, 25 mmol/L Tris (pH 7.4). The homogenate was brought to 20 mL with the same buffer followed by centrifugation at 6 × 10^2^ *g* for 10 min at 4°C to remove intact cells. The resulting supernatant was recentrifuged at 18 × 10^3^ *g* for 45 min at 4°C and the pellet obtained was resuspended in some of the supernatant and adjusted to 0.1 mol/L in NaCl and 0.2 mmol/L in MgSO_4_ before centrifugation at 30 × 10^3^ *g* for 30 min at 4°C. The pellet was then washed three times by resuspension in 300 mL of 25 mmol/L Tris (pH 7.4) and harvested by centrifugation at 20 × 10^2^ *g* for 30 min at 4°C. The membranes were finally suspended in 100 mmol/L imidazole buffer, pH 7.4, divided into 1.5 mL samples and stored at −20°C for further experimentation.

### Cell culture preparation

Human kidney‐2 (HK2) cells, which are immortalized human renal proximal tubular epithelial cells, were obtained from ATCC (Rockville, MD). The cells were cultured in RPMI‐1640 medium (Invitrogen, Grand Island, NY) supplemented with 10% heat‐inactivated fetal calf serum (Invitrogen) and 1% streptomycin/penicillin (Invitrogen), pH 7.4. They were plated at a density of 2.5 × 10^5^ cells/well in six‐well plates. Cell cultures were fed in every 48 h. Before conducting the experiments on ONOO^−^ reactivity, the cells were starved by serum‐free medium for 24 h. The viability of the cells was measured by the trypan blue exclusion assay.

### Plasma membrane fraction isolation from HK2 cells

The crude plasma membrane fractions were isolated from HK2 cells following the method of Holthouser et al. (2010). Postincubation, the treated cells were washed twice with PBS and placed in ice‐cold lysis buffer containing 50 mmol/L mannitol, 5 mmol/L Tris·HCl, pH 7.4, 10 *μ*L/mL phosphatase inhibitor cocktail, and 10 *μ*L/mL protease inhibitor cocktail. The lysates were then homogenized followed by centrifugation at 3.10^3^ *g* at 4°C for 15 min to remove cell debris. The supernatant was collected and centrifuged at 30.10^3^ *g* for 30 min. The pellet was resuspended in 100 mmol/L imidazole buffer, pH 7.4, and aliquots were preserved at −20°C for further experimentation.

### Measurement of NKA activity

NKA activity in renal plasma membrane fractions and HK2 cells (resuspended in 100 mmol/L imidazole buffer, pH 7.4) was measured by the method used by Mallick et al. (2000). This method was established in the 1980s (Gonçalves Moraes V. L. 1982) and has been widely used in many studies (Boldyrev et al. 1997; Mallick et al. 2000; Chakraborty et al. 2003; Holthouser et al. 2010) that provide the measure of NKA activity with reasonable accuracy. Briefly, an aliquot of the membrane suspension (100 *μ*L containing 100–250 *μ*g protein) was added to a reaction mixture containing 100 mmol/L NaCl, 10 mmol/L KCl, 6 mmol/L MgCl_2_, and 3 mmol/L ATP in 100 mmol/L imidazole buffer, pH 7.4, in the presence or absence of 3 mmol/L ouabain and incubated for 15 min at 37°C. These concentrations of NaCl, KCl, MgCl_2_, and ATP are typical for this assay and allow the pump to be saturated with Na^+^ and K^+^. The inorganic phosphate (Pi) liberated was measured and the enzyme expressed as *μ*moles of Pi liberated/mg protein/h. The ouabain‐sensitive ATPase activity was used as the measure of NKA.

### Determination of ONOO^−^ reactivity at different concentrations

Isolated renal plasma membrane fractions and HK2 cells were incubated for 30 min at 37°C in the presence or absence of various concentrations of ONOO^−^ ranging from 10 nmol/L to 200 *μ*mol/L. At the end of the incubation period, NKA activity in these membrane or cell preparations was measured as mentioned earlier. To determine the specificity of ONOO^−^‐induced responses, the membrane fractions or cell preparations were pretreated with or without a ONOO^−^ scavenging agent, MEG (200 *μ*mol/L, Enzo Life Sciences) for 10 min at 37°C. Following pretreatment, the membranes were incubated for 30 min at 37°C in the presence or absence of various concentrations of ONOO^−^ ranging from 10 nmol/L to 200 *μ*mol/L. To determine whether NO or O_2_^−^ has any direct role in the responses induced by ONOO^−^, the membrane fractions or cell preparations were pretreated either with l‐NAME (100 *μ*mol/L, Sigma) or TEMPOL (1 mmol/L, Sigma) for 10 min at 37°C. At the end of pretreatment, the cells were incubated for 30 min at 37°C in the presence or absence of various concentrations of ONOO^−^ ranging from 500 pmol/L to 200 *μ*mol/L.

## Results

### Effect of different time period incubation of ONOO^−^ solutions on NKA activity

**Figure 1:**
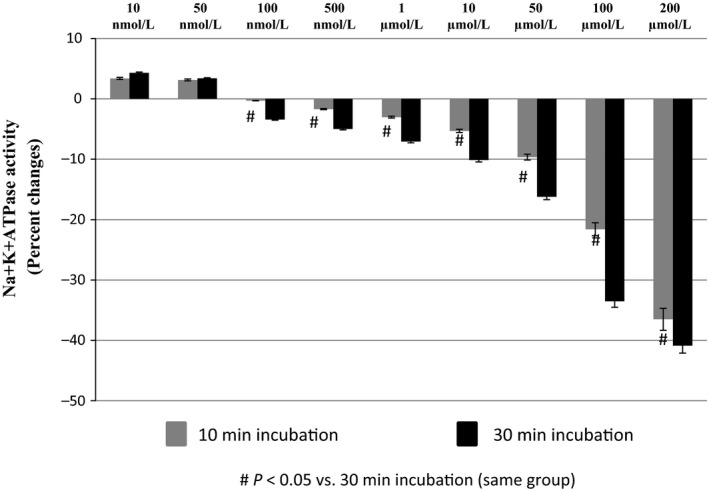
Peroxynitrite (ONOO ^−^)‐induced changes in Na^+^K^+^ ATPase (NKA) activity during 10 and 30 min of incubation periods with ONOO ^−^ solutions in renal plasma membrane fraction preparations (*n *=* *5 in each group).

NKA activity (ouabain‐inhibitable ATPase activity) was determined in plasma membrane fractions (obtained from mouse renal tissues and HK2 cells) after incubation with various concentrations of ONOO^−^ for 10 and 30 min of incubation at 37°C. The baseline values of NKA activity in the renal plasma membrane fractions was measured as 14.52 ± 0.29 *μ*mol/L of Pi liberated/mg protein/h. Figure 1 shows no significant difference in the stimulatory effect of ONOO^−^ at lower concentrations (10 nmol/L and 50 nmol/L) between 10 and 30 min of incubation. However, a significant difference in NKA activity between 10 and 30 min of incubation was observed at higher concentrations (0.30 ± 1.15% vs. −3.43 ± 0.38% of basal activity at 100 nmol/L to −21.60 ± 1.28% vs. −33.51 ± 2.20% of basal activity at 100 *μ*mol/L ONOO^−^ concentration). At 200 *μ*mol/L ONOO^−^ concentration, the difference in NKA activity between 10 and 30 min of incubation was less (decreased to −36.51 ± 1.85% vs. −40.88 ± 1.20% of basal activity), but this difference was also found to be statistically significant (Fig. 1). The average difference in NKA activity between 10 and 30 min of incubation from 100 nmol/L to 200 *μ*mol/L of ONOO^−^ concentration was found to be 37% (Fig. 1). It is clear that at 30 min of incubation, in comparison to exposure from lower concentrations, higher concentrations of ONOO^−^ had significantly more impact on NKA activity compared to corresponding concentrations of ONOO^−^ at 10 min of incubation time. Given the reactivity and short half‐life of ONOO^−^, it was expected that incubation for a longer period would reduce the reactivity of ONOO^−^ as it was degraded. However, it showed that the 30‐min incubation period caused minimal changes in the reactivity of ONOO^−^ on NKA activity. Thus, in all the following experimental protocols involving incubation with ONOO^−^ in the presence and absence of different free radical scavengers, a 30‐min incubation time was utilized.

### Effect of ONOO^−^ treatment on NKA activity in the presence of MEG

**Figure 2:**
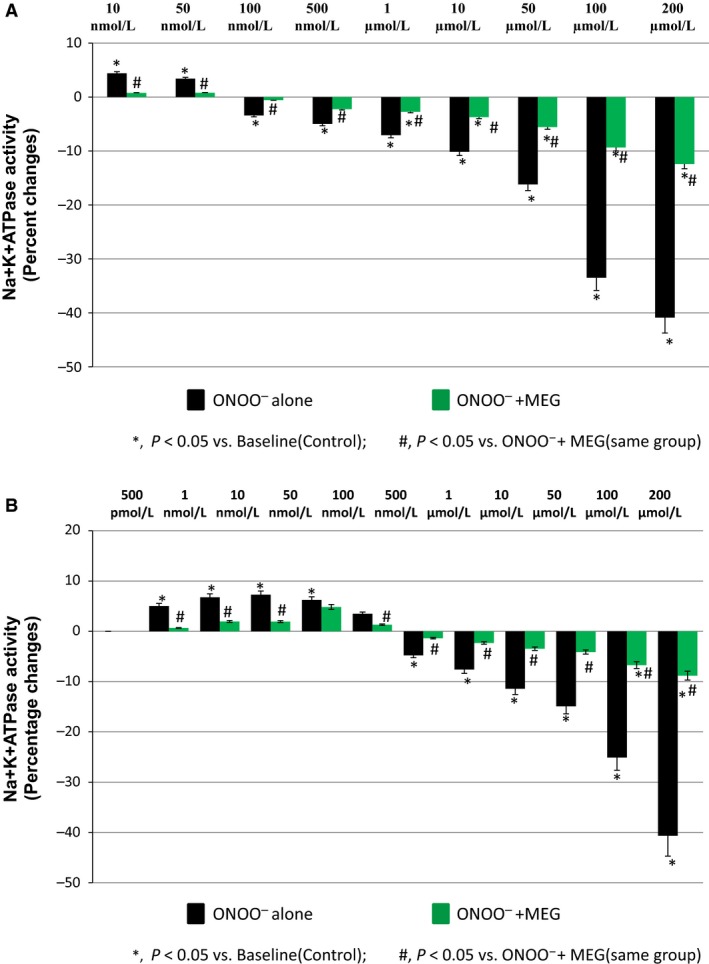
Peroxynitrite (ONOO ^−^)‐induced changes in Na^+^K^+^ ATPase (NKA) activity in the presence of mercaptoethylguanidine (MEG; 200 *μ*mol/L) in (A) renal plasma membrane fraction preparations and (B) HK2 cell preparations (*n *=* *5 in each group).

The role of ONOO^−^ on NKA activity was examined using 2‐mercaptoethylguanidine (MEG), a known scavenger of ONOO^−^ (Cuzzocrea et al. 1998) in the presence and absence of ONOO^−^ in renal plasma membrane fraction at 30 min of incubation at 37°C. MEG at a fixed concentration of 200 *μ*mol/L was preincubated with renal plasma membrane fraction for 10 min before the addition of various concentrations of ONOO^−^ (ranging from 10 nmol/L to 200 *μ*mol/L) at 37°C for 30 min. Interestingly, MEG treatment markedly attenuated these ONOO^−^‐induced changes in NKA activity, both at lower concentrations (activity reduction of 4.38 ± 0.20% to 0.75 ± 0.27% of basal activity at 10 nmol/L onward to 3.40 ± 0.28% to 0.78 ± 0.24% of basal activity at 50 nmol/L; an average activity reduction of 79 ± 3.4% from maximal stimulation at 100%) and at higher concentrations (activity reduction of −3.43 ± 0.38% to −0.57 ± 0.64% of basal activity at 100 nmol/L onward to −40.88 ± 1.21% to −12.43 ± 0.81% of basal activity at 200 *μ*mol/L; an average activity reduction of 67 ± 5.5% from maximal inhibition at 100%) (Fig. 2A).

In HK2 cells, a similar attenuating effect of MEG on ONOO^−^‐induced changes in NKA activity was observed, blocking the stimulatory effect of ONOO^−^ at lower concentrations and inhibitory effect at higher concentrations. The baseline values of NKA activity in the HK2 cells was measured as 10.96 ± 0.32 *μ*moles of Pi liberated/mg protein/h. In HK2 cells, MEG treatment markedly attenuated these ONOO^−^‐induced changes in NKA activity, both at lower concentrations (activity reduction of 5.28 ± 0.51% to 0.50 ± 0.28% of basal activity at 500 pmol/L onward to 1.95 ± 0.65% to 1.25 ± 0.25% of basal activity at 100 nmol/L; an average activity reduction of 65 ± 4.3% from maximal stimulation at 100%) and at higher concentrations (activity reduction of −5.32 ± 0.69% to −1.02 ± 0.40% of basal activity at 500 nmol/L onward to −41.74 ± 1.32% to −8.36 ± 1.63% of basal activity at 200 *μ*mol/L; an average activity reduction of 78 ± 4.7% from maximal inhibition at 100%) (Fig. 2B).

### Effect of ONOO^−^ treatment on NKA activity in the presence of TEMPOL

**Figure 3:**
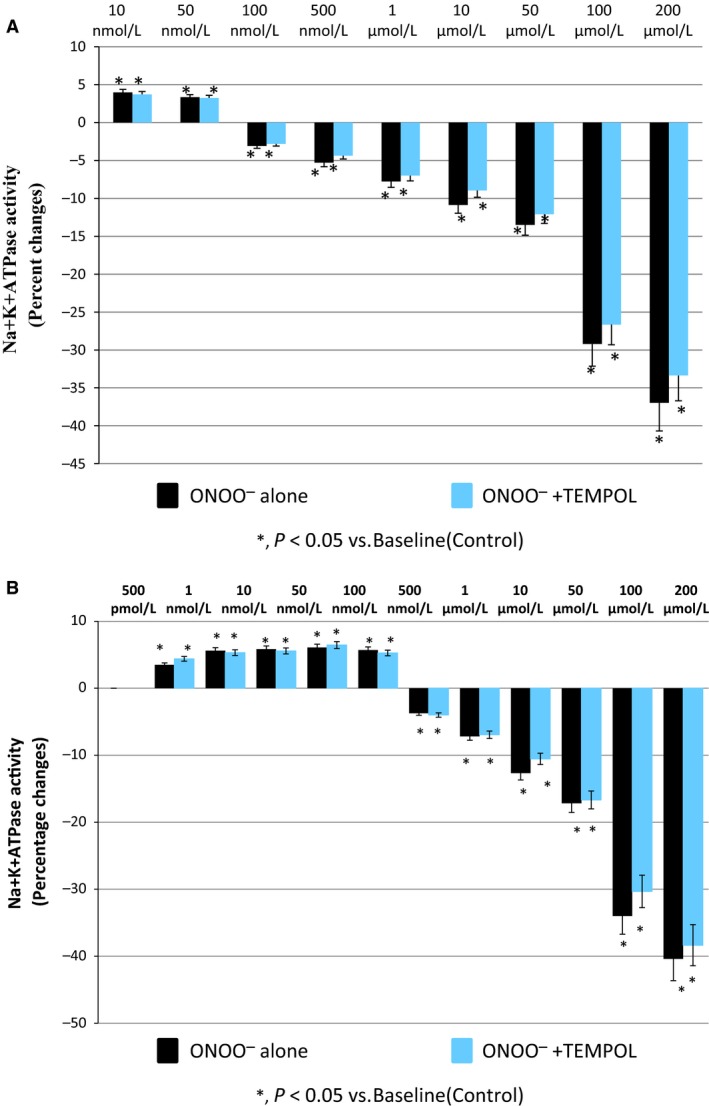
Peroxynitrite (ONOO ^−^)‐induced changes in Na^+^K^+^ ATPase (NKA) activity in the presence of superoxide scavenger, TEMPOL (1 mM) in (A) renal plasma membrane fraction preparations and (B) HK2 cell preparations (*n *=* *5 in each group).

To elucidate any involvement of superoxide (O_2_^−^) radical in ONOO^−^‐induced changes in NKA activity, TEMPOL, a scavenger of superoxide (Majid et al. 2005) was used in the presence and absence of ONOO^−^ in both renal plasma membrane and HK2 cells at 30‐min incubation. TEMPOL at a fixed concentration of 1 mmol/L was preincubated with renal plasma membrane fractions for 10 min before the addition of various concentrations of ONOO^−^ (ranging from 10 nmol/L to 200 *μ*mol/L) at 37°C for 30 min. Interestingly, TEMPOL treatment did not significantly attenuate these ONOO^−^‐induced changes in NKA activity, both at lower concentrations (3.97 ± 0.28% vs. 3.72 ± 0.27% of basal activity at 10 nmol/L to 3.35 ± 0.28% vs. 3.26 ± 0.24% of basal activity at 50 nmol/L; an average activity reduction of only 4.5 ± 1.3% from maximal stimulation at 100%) and at higher concentrations (−3.09 ± 0.25% vs. −2.82 ± 0.30% of basal activity at 100 nmol/L to −36.9 ± 0.94% vs. −33.37 ± 1.18% of basal activity at 200 *μ*mol/L; an average activity reduction of only 11.8 ± 0.8% from maximal inhibition at 100%) (Fig. 3A).

A similar trend of results was also observed in HK2 cells with the same incubation protocols. Both at lower concentrations (3.58 ± 0.55% vs. 4.29 ± 0.23% of basal activity at 500 pmol/L to 5.78 ± 1.12% vs. 5.19 ± 1.15% of basal activity at 100 nmol/L; an average activity reduction of only 8.55 ± 0.75% from maximal stimulation at 100%) and at higher concentrations (−3.55 ± 1.14% vs. −4.0 ± 0.61% of basal activity at 500 nmol/L to −40.42 ± 1.98% vs. −38.36 ± 2.10% of basal activity at 200 *μ*mol/L; an average activity reduction of only 5.64 ± 0.6% from maximal inhibition at 100%) of ONOO^−^ treatment, TEMPOL showed no blocking effect on the effects of ONOO^−^ on NKA activity (Fig. 3B).

### Effect of ONOO^−^ treatment on NKA activity in the presence of l‐NAME

**Figure 4:**
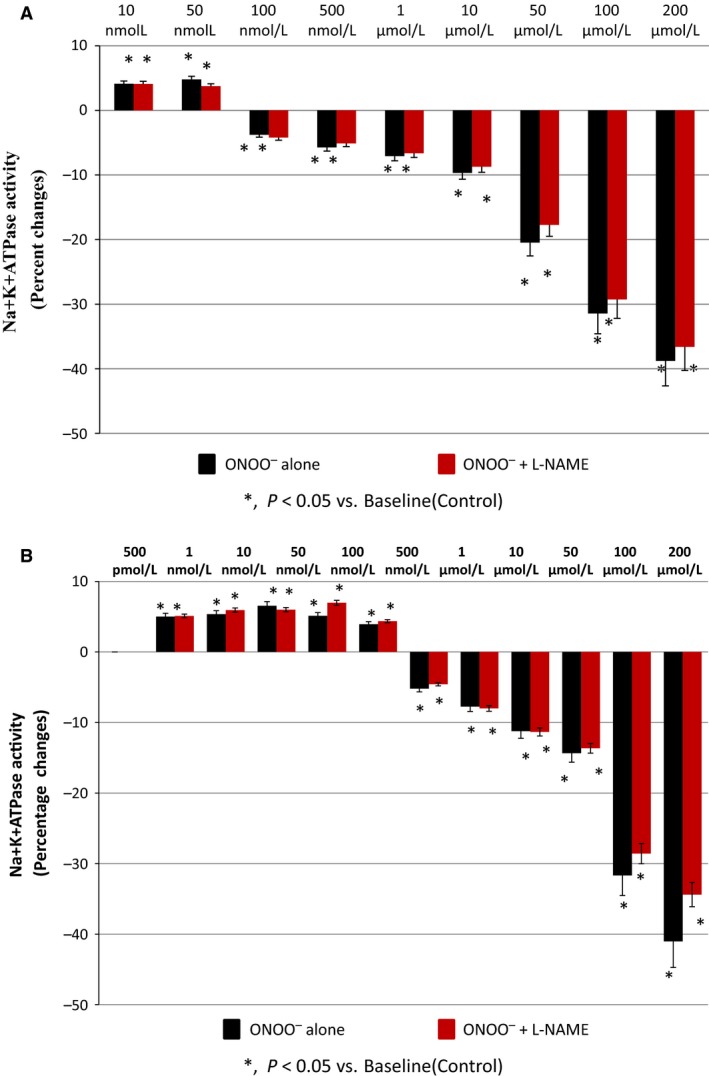
Peroxynitrite (ONOO ^−^)‐induced changes in Na^+^K^+^ ATPase (NKA) activity in the presence of NO synthase inhibitor, l‐NAME (100 *μ*mol/L) in (A) renal plasma membrane fraction preparations and (B) HK2 cell preparations (*n *=* *5 in each group).

To further examine the role of nitric oxide (NO) in the ONOO^−^‐induced changes in NKA activity at both lower and higher concentrations, an inhibitor of NO synthase enzyme, l‐NAME (Majid et al. 2005) was used in this study at a fixed concentration of 100 *μ*mol/L. At lower concentrations (4.13 ± 0.16% vs. 4.06 ± 0.27% of basal activity at 10 nmol/L to 4.76 ± 0.28% vs. 3.67 ± 0.24% of basal activity at 50 nmol/L; an average activity reduction of only 12.29 ± 0.9% from maximal stimulation at 100%) and at higher concentrations (−3.65 ± 0.70% vs. −4.12 ± 0.52% of basal activity at 100 nmol/L to −38.93 ± 0.81% vs. −36.40 ± 2.0% of basal activity at 200 *μ*mol/L; an average activity reduction of only 9.21 ± 0.8% from maximal inhibition at 100%) of ONOO^−^ treatment, l‐NAME showed no blocking on the effects of ONOO^−^ on NKA activity (Fig. 3A) (Fig. 4A).

Similar results were also observed in HK2 cells, no attenuating impact of l‐NAME was observed at lower concentrations (5.27 ± 0.39% vs. 5.46 ± 0.51% of basal activity at 500 pmol/L to 4.21 ± 0.26% vs. 3.77 ± 0.65% of basal activity at 100 nmol/L; an average activity reduction of only 9.46 ± 0.77% from maximal stimulation at 100%) and at higher concentrations (−5.11 ± 0.53% vs. −4.94 ± 0.52% of basal activity at 500 nmol/L to −41.95 ± 1.11% vs. −34.04 ± 1.66% of basal activity at 200 *μ*mol/L; an average activity reduction of only 5.03 ± 0.6% from maximal inhibition at 100%)(Fig. 4B).

## Discussion

In the present study, the experimental data show that there is a dose‐dependent biphasic action of ONOO^−^ on NKA activity causing stimulation of NKA activity at lower concentrations and inhibition at higher concentrations. These ONOO^−^‐induced responses on NKA activity were attenuated by pretreatment with MEG confirming that these responses are specific to ONOO^−^. These findings implicate that ONOO^−^ acts as a signaling component in the regulation of NKA activity. As normal plasma ONOO^−^ level is within nanomolar range (Radi et al. 2002; Nalwaya and Deen 2005; Quijano et al. 2005; Szabó et al. 2007; Matavelli et al. 2009), these findings strongly suggest that ONOO^−^ formation in normal conditions has a considerable role as a stimulator of NKA activity that may help to maintain normal tubular sodium reabsorptive function. Such maintenance of sodium homeostasis is an important protective function served by ONOO^−^ at the physiological state as it is continually formed in tissues by the interactions of endogenously formed NO and O_2_^−^ in the biological tissues (Majid et al. 2005). Inhibiting NKA activity at high concentrations of ONOO^−^, achieved in various pathophysiological conditions of enhanced oxidative stress, also indicates a protective role of ONOO^−^ such as to prevent excessive sodium retention in such conditions. Thus, these biphasic actions of endogenous ONOO^−^ formation may implicate an important protective role for this radical compound in maintaining sodium homeostasis both in physiological as well as in pathophysiological conditions associated with oxidative stress.

Previously, it has been reported that unlike the fast forward reaction of O_2_^−^ and NO generating ONOO^−^, the slow reverse reaction of ONOO^−^ forming O_2_^−^ and NO is also possible in biological conditions. In fact, when the productions of O_2_^−^ and NO occur at very low levels, the reverse reaction can be significant (Pacher et al. 2007). In addition to such reverse reaction of ONOO^−^ forming O_2_^−^ and NO in biological conditions, it has also been reported that the NO synthase also acts as a nitrite reductase to reduce nitrite to NO (Gautier et al. 2006; Matavelli et al. 2009). Another source of NO generation from ONOO^−^ may involve the major end product of ONOO^−^ production, the anion nitrite, which can also be reduced to NO (Gautier et al. 2006; Matavelli et al. 2009). However, we observed no significant attenuating effect of TEMPOL or l‐NAME pretreatment on the NKA activity induced by ONOO^−^ on both lower and higher concentrations in renal plasma membrane fractions or in HK2 cells (Figs 3 and 4). These findings demonstrate that neither O_2_^−^ nor NO has any direct role in the signaling mechanisms induced by ONOO^−^ on renal NKA activity. However, the increases in NKA activity due to a low dose of ONOO^−^ could be caused by activation of mitogen‐activated protein kinase (MAPK) as it was reported earlier that ONOO^−^ activates MAPK in rat fibroblasts (Bapat et al. 2001 Jun 15). It is also reported that the enhancement in NKA activity at low doses of angiotensin II (AngII) administration (which could cause ONOO^−^ generation by enhancing both NO and O_2_^−^ formation) is mediated by MAPK activation (Banday and Lokhandwala 2008). Although in the same study (Banday and Lokhandwala 2008), it was also shown that the NO‐cGMP signaling pathway was involved in NKA inhibition with high dose of AngII treatment in proximal tubular cells, this is not seen in the present study as pretreatment with neither l‐NAME nor TEMPOL significantly altered the inhibitory effects of ONOO^−^ on NKA. As MEG (ONOO^−^ scavenger) abolishes this inhibitory effect of ONOO^−^ on NKA enzyme, it may be possible that a NO‐independent activation of soluble guanylate cyclase activation may induce such an inhibitory effect on NKA by high doses of ONOO^−^ (Korkmaz S et al. 2013). Future comprehensive experiments would be needed to examine the signaling pathways involved in these actions of ONOO^−^ in modulating NKA activity.

The implication of these findings in vitro, such as the stimulation and the inhibition of NKA at low‐ and high‐ONOO^−^ concentrations may be of immense importance in terms of renal function in vivo. NKA stimulation by low concentration of ONOO^−^ may help this enzyme to maintain the electrochemical gradient in the cell membrane maintaining a higher concentration of sodium outside than inside while maintaining a higher concentration of potassium inside the cell than outside. Functional NKA activity mediates a significant part of tubular sodium reabsorptive function in the kidney. These findings confirm that ONOO^−^ at nanomolar concentrations (normal levels in the plasma) has a considerable role as a stimulator of NKA activity that helps to maintain normal tubular sodium reabsorptive function. When the NKA activity is inhibited by high‐ONOO^−^ concentration, it may affect sodium reabsorption in the kidney similarly as noticed in the application of inhibitory concentrations of ouabain where nearly half of the sodium reabsorption is inhibited (Katz 1982). Besides, the normal or stimulated activity of NKA induced by low‐ONOO^−^ concentration may promote not only the primary active transport of these cations, but also indirectly various secondary active transport processes (like Ca^2+^ extrusion by Na‐Ca exchange, reabsorption of Na^+^ in the ascending limb of Henle's loop) that may be markedly affected by high concentration of ONOO^−^. As the NKA activity is abundant in the kidney and a greater part of its energy consumption is invested in the reabsorption of sodium, it has become logical to assess whether there exists any regional variability in ONOO^−^‐induced NKA activity in the kidney in future experiments.

The mechanisms by which ONOO^−^ regulates NKA activity will be of immediate interest as well in future experiments even though some studies of similar nature have already been carried out (Zhang et al. 2002; Szabó et al. 2007; Reifenberger et al. 2008; Liu J et al. 2012). Proposed mechanisms of inhibition of NKA activity include direct peroxidative damage on NKA (like nitration of protein tyrosine, interactions with NKA‐SH groups, changes in membrane fluidity, etc.) in addition to known signaling pathways (like Src, MAPK/ERK‐1/2, Akt, etc.) that can affect the normal functioning of NKA, however, these pathways are not always clear and conclusive (Zhang et al. 2002; Szabó et al. 2007; Reifenberger et al. 2008). It will also be interesting to elucidate the involvement of the signaling pathways involving various protein kinases in the stimulation/inhibition of NKA activity induced by various concentrations of ONOO^−^.

In the present study, we have not investigated the conformational changes in NKA enzyme (Morth et al. 2007) that may possibly occur during ONOO^−^ treatment. However, it was shown in a previous study (Figtree et al. 2009) that ONOO− at a concentration of 100 *μ*mol/L (equivalent to a high dose used in the present study) induced glutathionylation of the NKA *β*1 subunit obtained from pig kidney tissue preparations. This was associated with a ≈twofold decrease in the rate‐limiting E2→E1 conformational change of the pump, as determined by RH421 fluorescence. In another study (Reifenberger et al. 2008), it was also shown that the ONOO^−^‐induced inhibition of NKA showed smaller changes in EPNa conformation than E(K) conformation, suggesting that the EPNa conformation of the pump is slightly more sensitive to ONOO^−^ than that in the E(K) conformation. However, these reported studies (Reifenberger et al. 2008; Figtree et al. 2009) were done with a high concentration of ONOO^−^ which inhibited the NKA activity. Thus, the effect of a low dose of ONOO^−^ (which increases the NKA activity) on the conformational changes in NKA activity remains to be examined in future studies. (Figtree et al. (2009)) also demonstrated that when the membranes isolated from pig kidney tissue preparations loaded with biotin‐GSH were exposed to solutions containing 100 *μ*mol/L of ONOO^−^, it did not change the *K*_m_ in the hydrolytic reaction of Na^+^ or K^+^ in NKA enzyme. However, the ONOO^−^/GSH lowered *V*_max_ in the hydrolytic activities of Na^+^ and K^+^ in NKA enzyme. It has also been shown in their study (Figtree et al. 2009) that both *K*_m_ and *V*_max_ in the hydrolytic activities by ATP were attenuated by ONOO^−^/GSH although GSH alone did not alter these parameters suggesting that ONOO^−^ at a high dose decreases the enzymatic activity. However, *K*_m_ and *V*_max_ of the NKA enzyme for the hydrolytic activity of ATP/Na^+^/K^+^ in the presence of a low dose of ONOO^−^ (which increases the NKA activity) remains to be examined in future studies.

In conclusion, our data suggest that ONOO^−^ exerts a biphasic action in the signaling mechanisms in regulating NKA activity. These data indicate that NKA activity remains unaffected or rather increased at the physiological concentration of ONOO^−^ which regulates tubular sodium reabsorption to maintain sodium hemostasis under normal conditions. On the other hand, NKA inhibition at high concentrations of ONOO^−^ may help to prevent excessive sodium retention in various pathophysiological conditions of enhanced oxidative stress. Thus, these findings implicate that endogenous ONOO^−^ formation serves an important protective role in maintaining sodium homeostasis both in the physiological as well as in the pathophysiological state associated with oxidative stress conditions.

## Conflict of Interest

None declared.
